# Supplementary material for: Dual Lineages of Langerhans Cells Cooperate to Restore the Immune Barrier after Skin Injury
Source: Adv Sci (Weinh). 2026 Jul 29;13(59):e76816. doi: 10.1002/advs.76816 (PMC13418046; doi:10.1002/advs.76816)
Supplement: Supplementary file 1 [file ADVS-13-e76816-s004.pdf]

Figure S1. Epithelial cell migration on day 2 after injury.

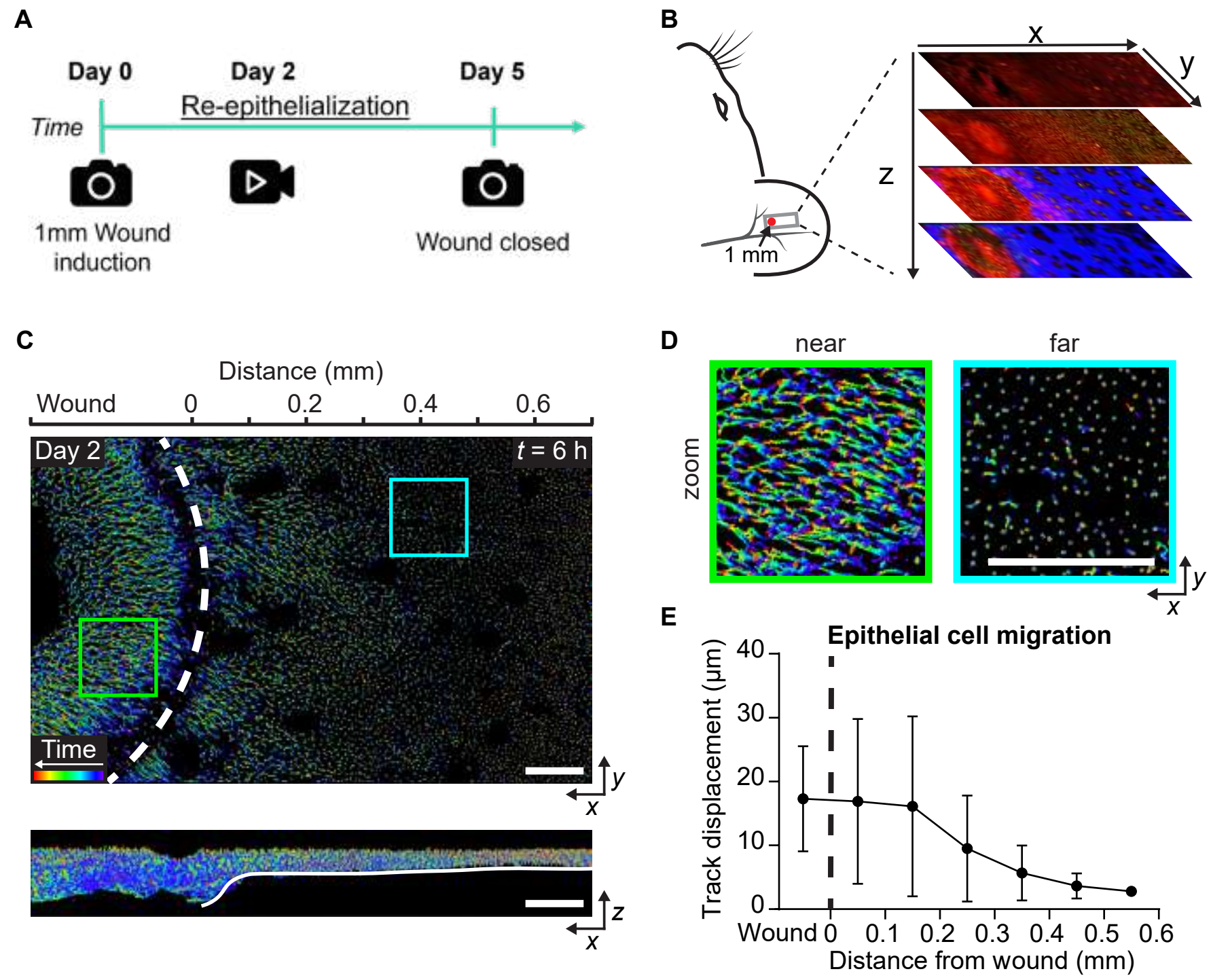

Figure S2. Tamoxifen-induced CreER recombination in vivo.

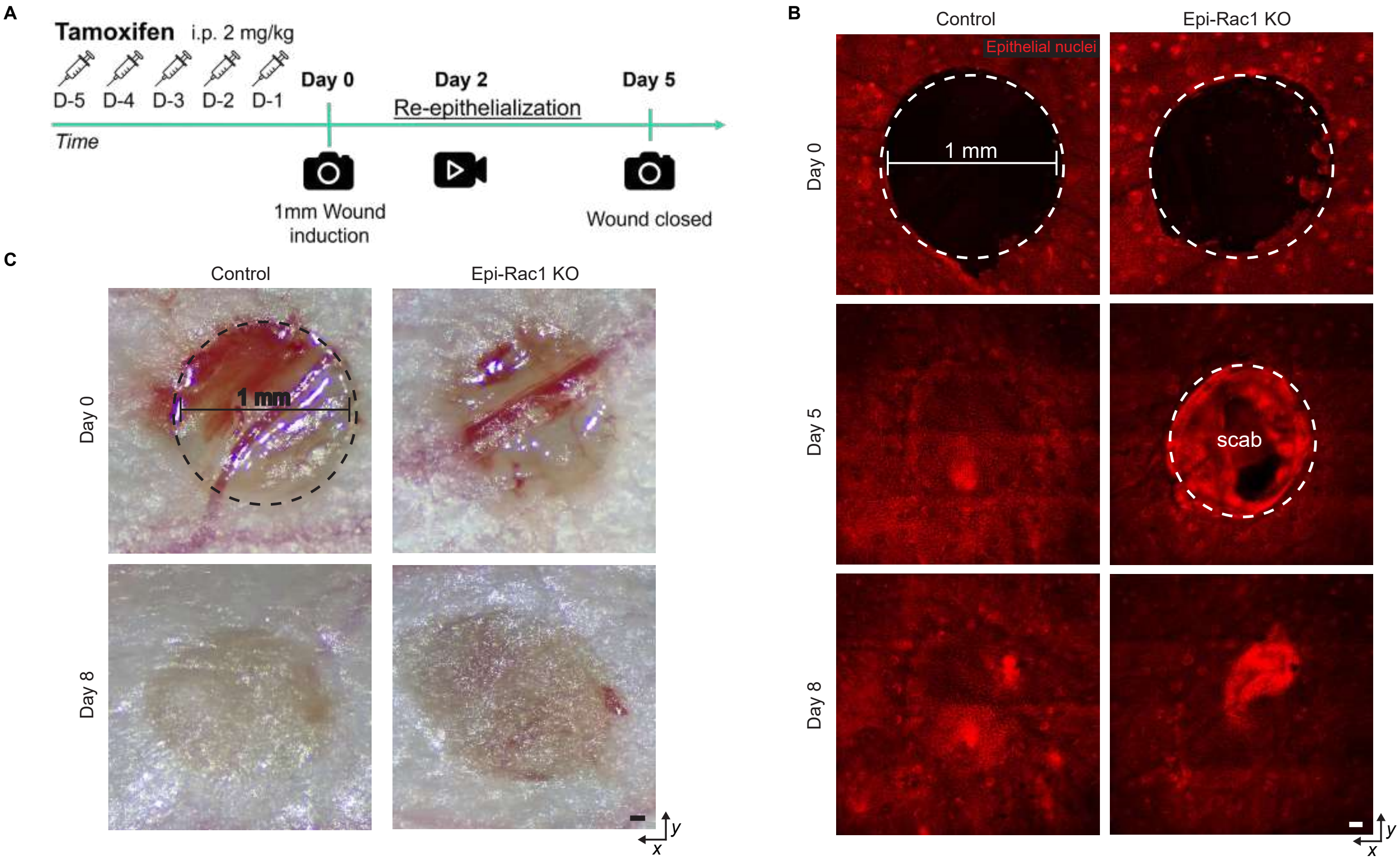

Figure S3. The majority of MHC-II^{+} cells in the wound epidermis are Langerhans cells.

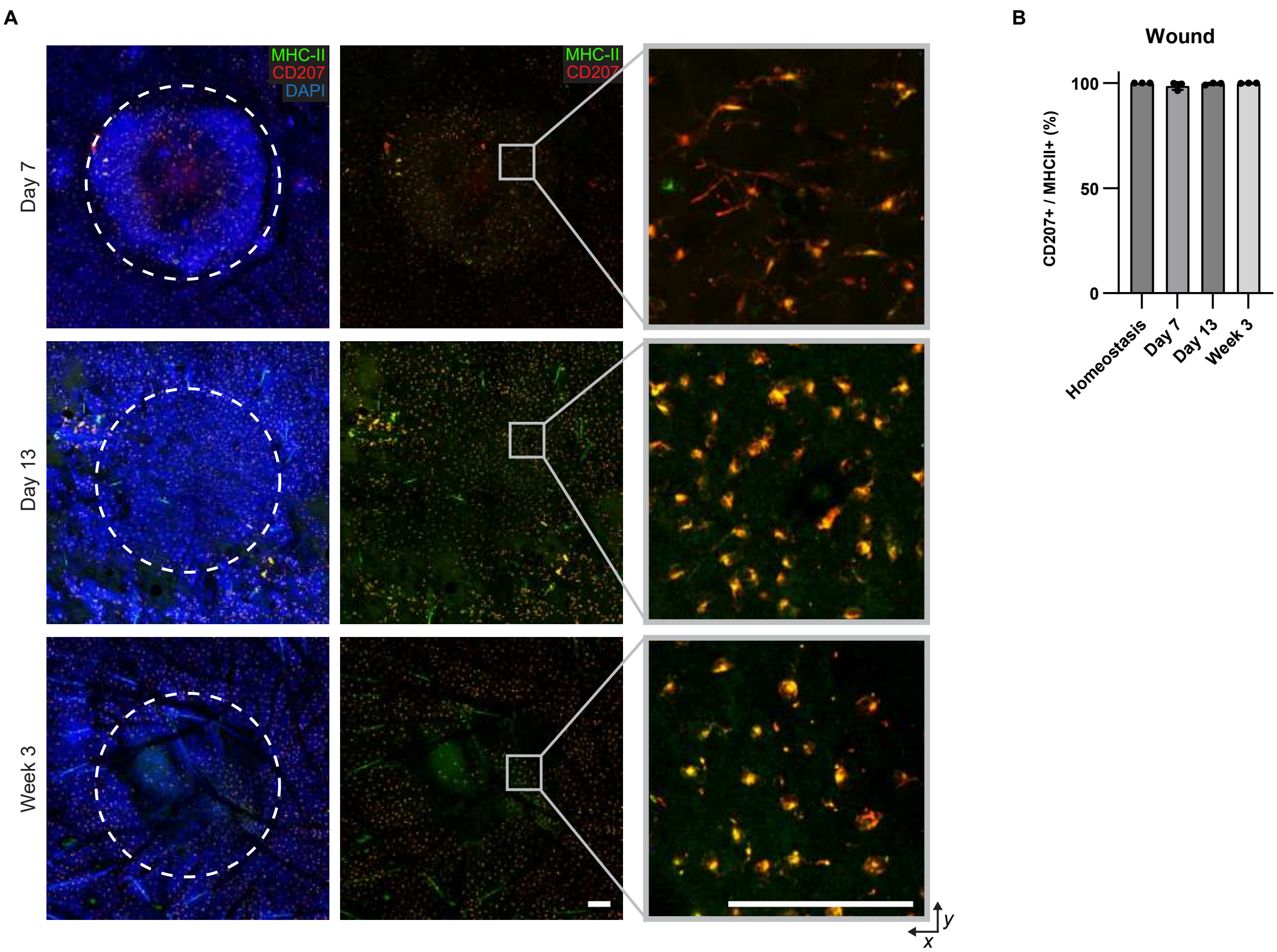

Figure S4. IL-34 is increased on day 7 of wound healing.

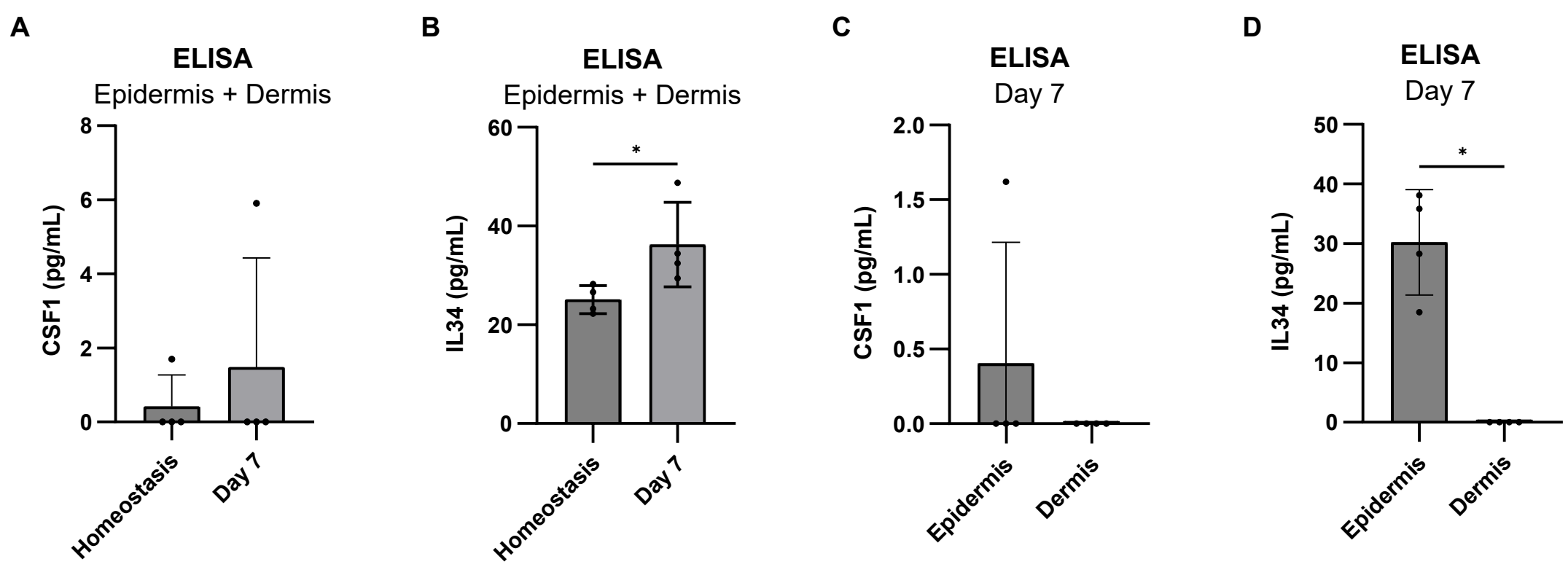

Figure S5. Langerhans cells quickly recover their normal distribution in the epidermal basal layer after wound induction.

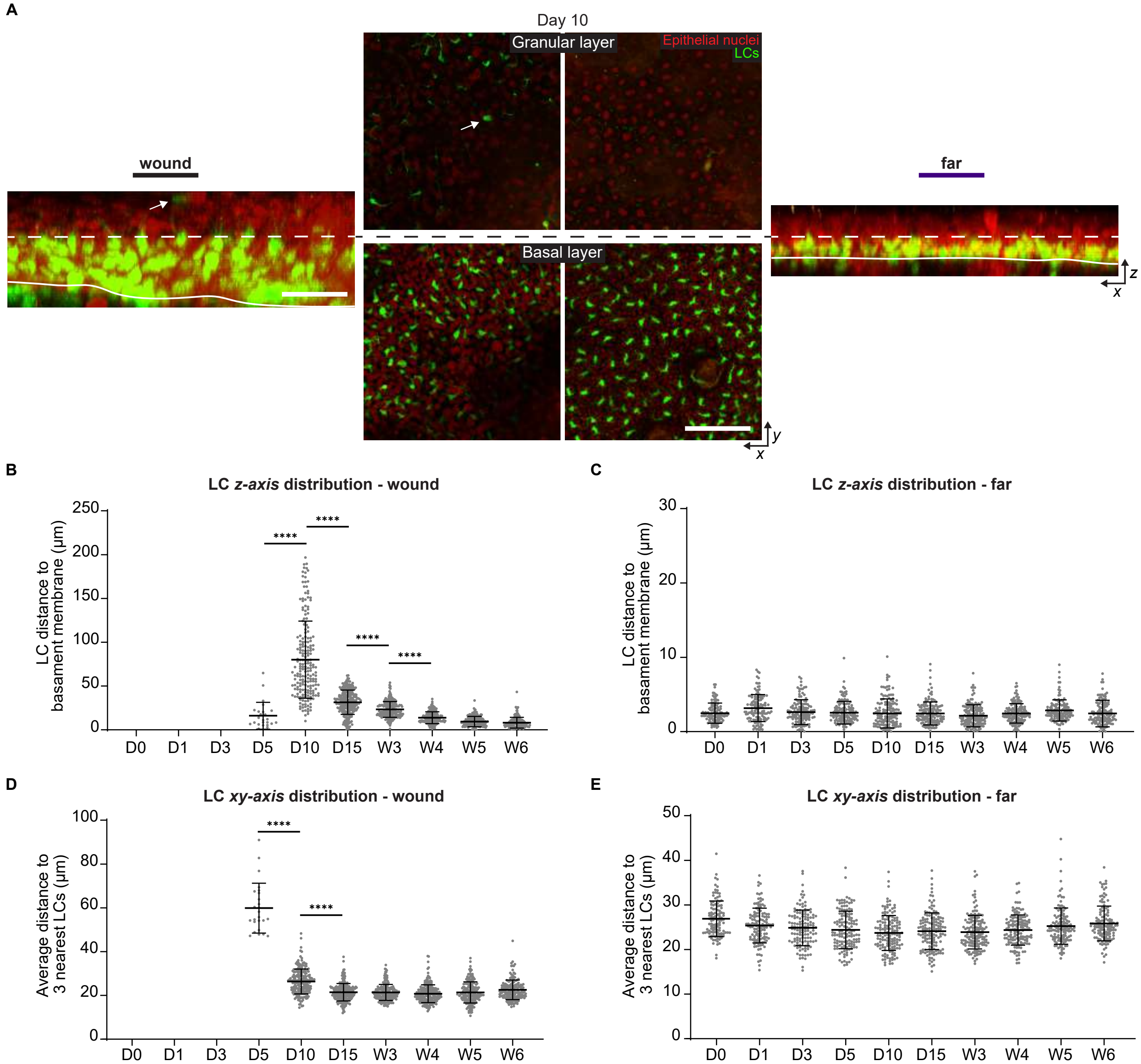

Figure S6. scRNA-Seq heterogeneity in the epidermis on day 11 after wound induction.

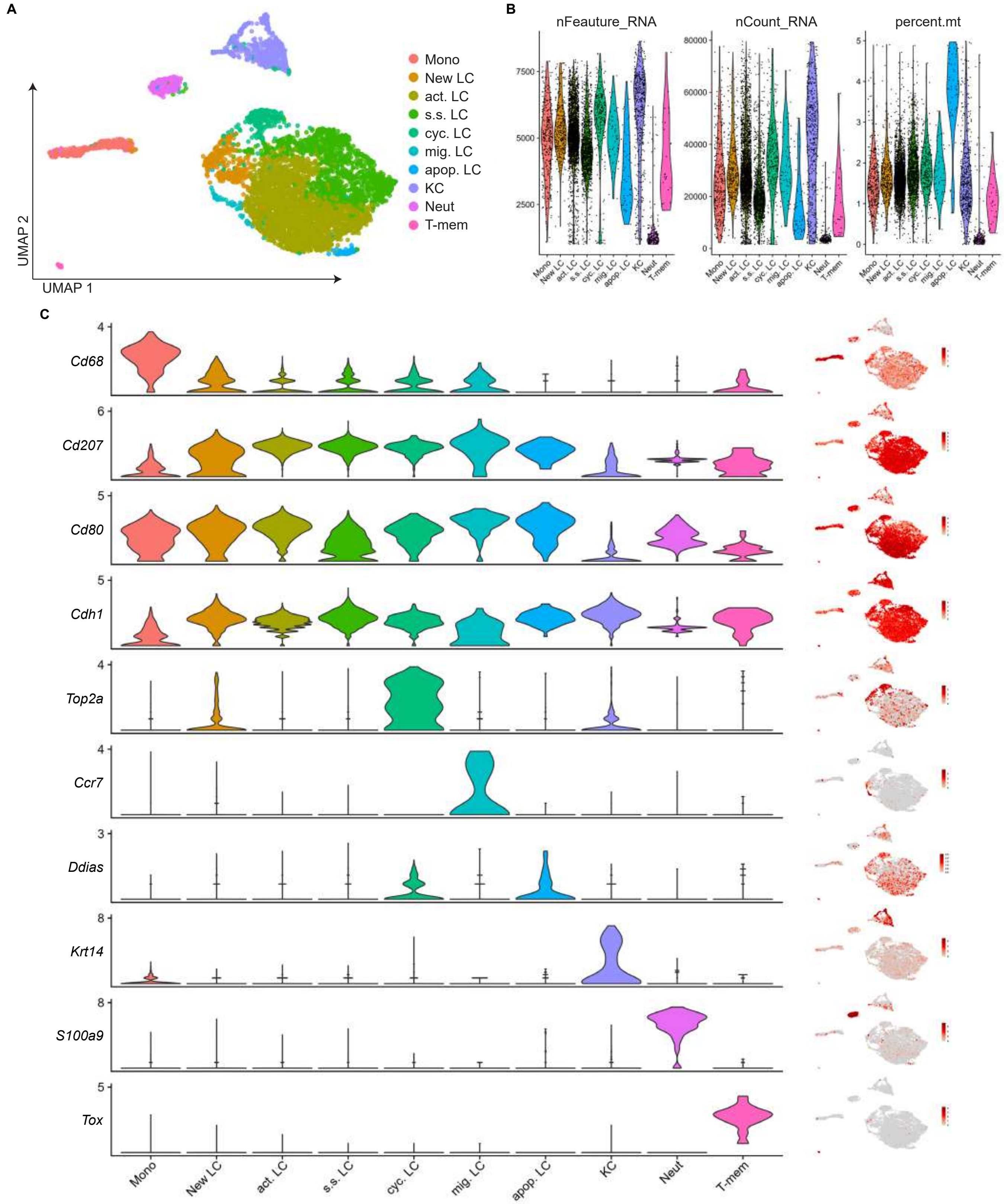

Figure S7. Steady-state vs activated eLC and GO terms gene expression.

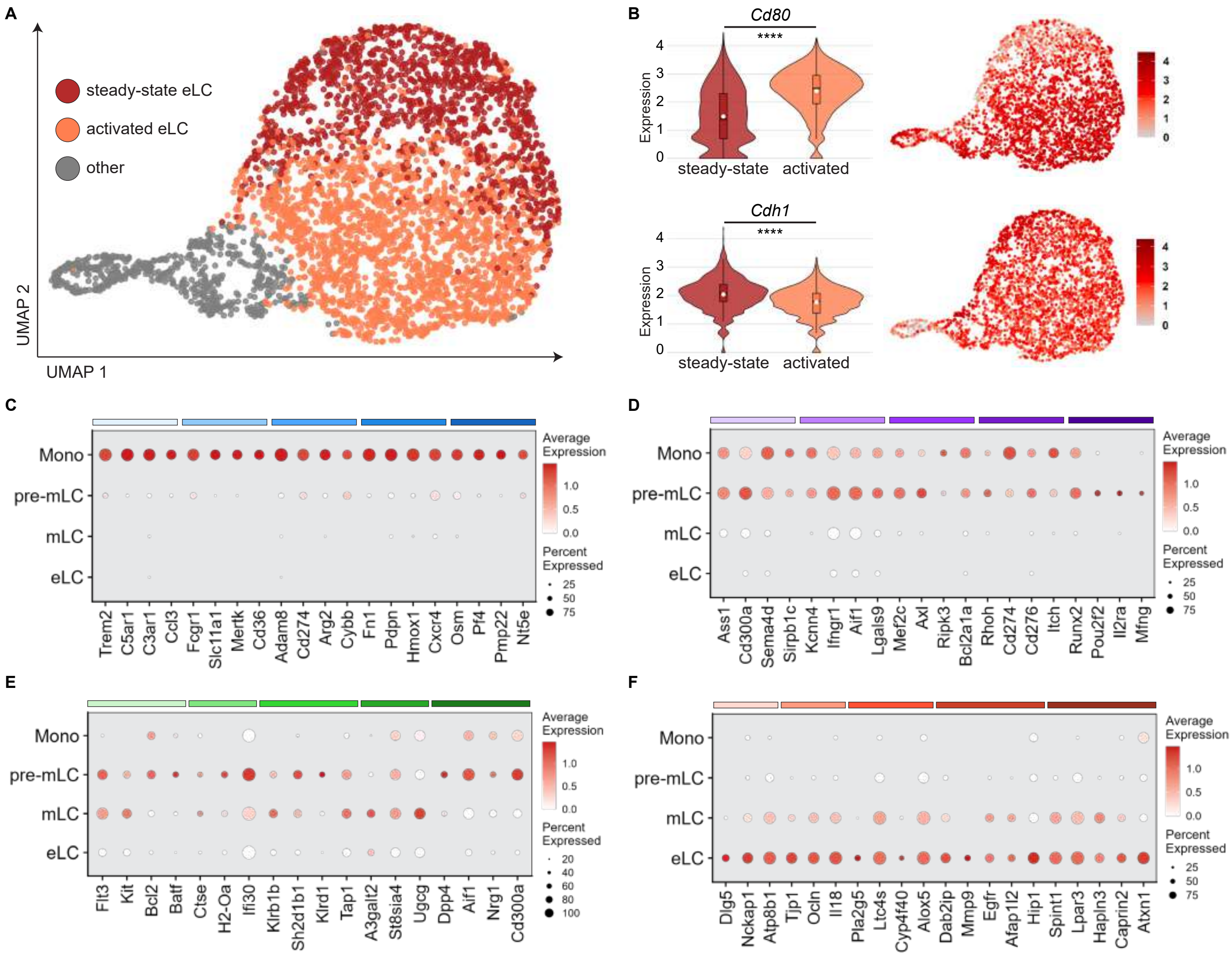

Figure S8. Adoptive transfer of monocytes give rise to Langerhans cells during wound healing.

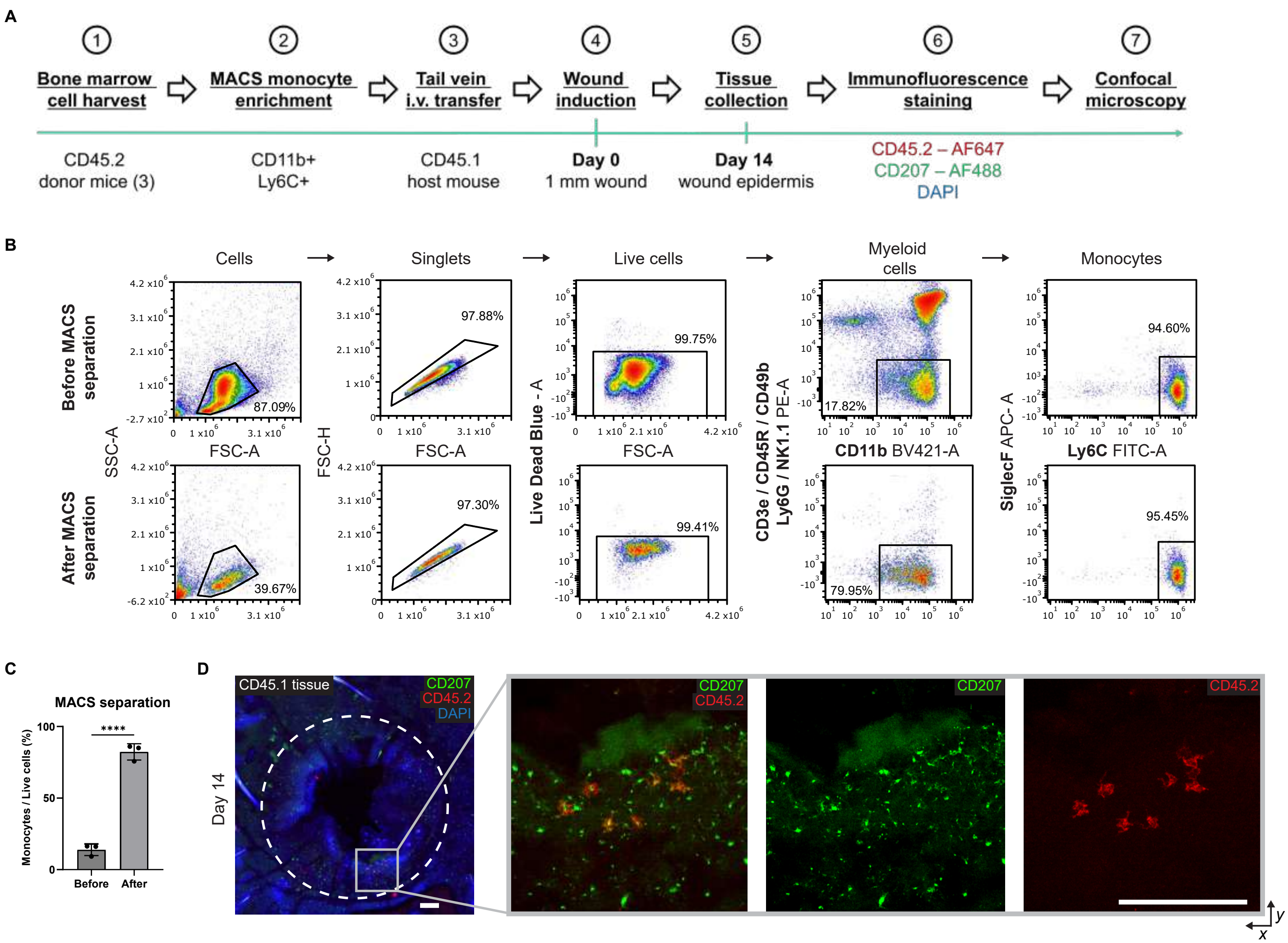

Figure S9. Characterization of epidermal CCR2-GFP cells at day 13 of wound healing.

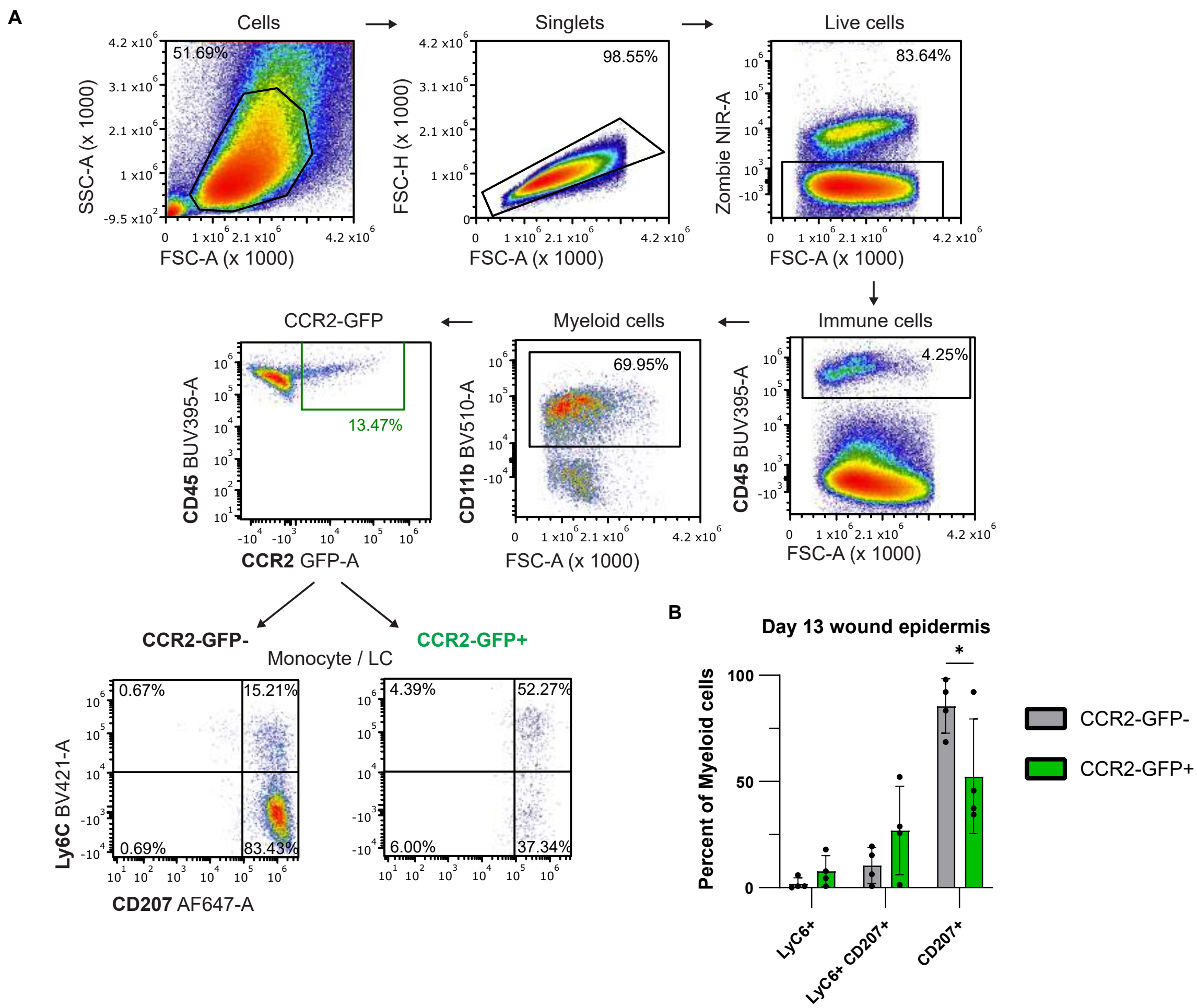

Figure S10. CXCR4 and CCR7 are not required for Langerhans cells migration during re-epithelialization.

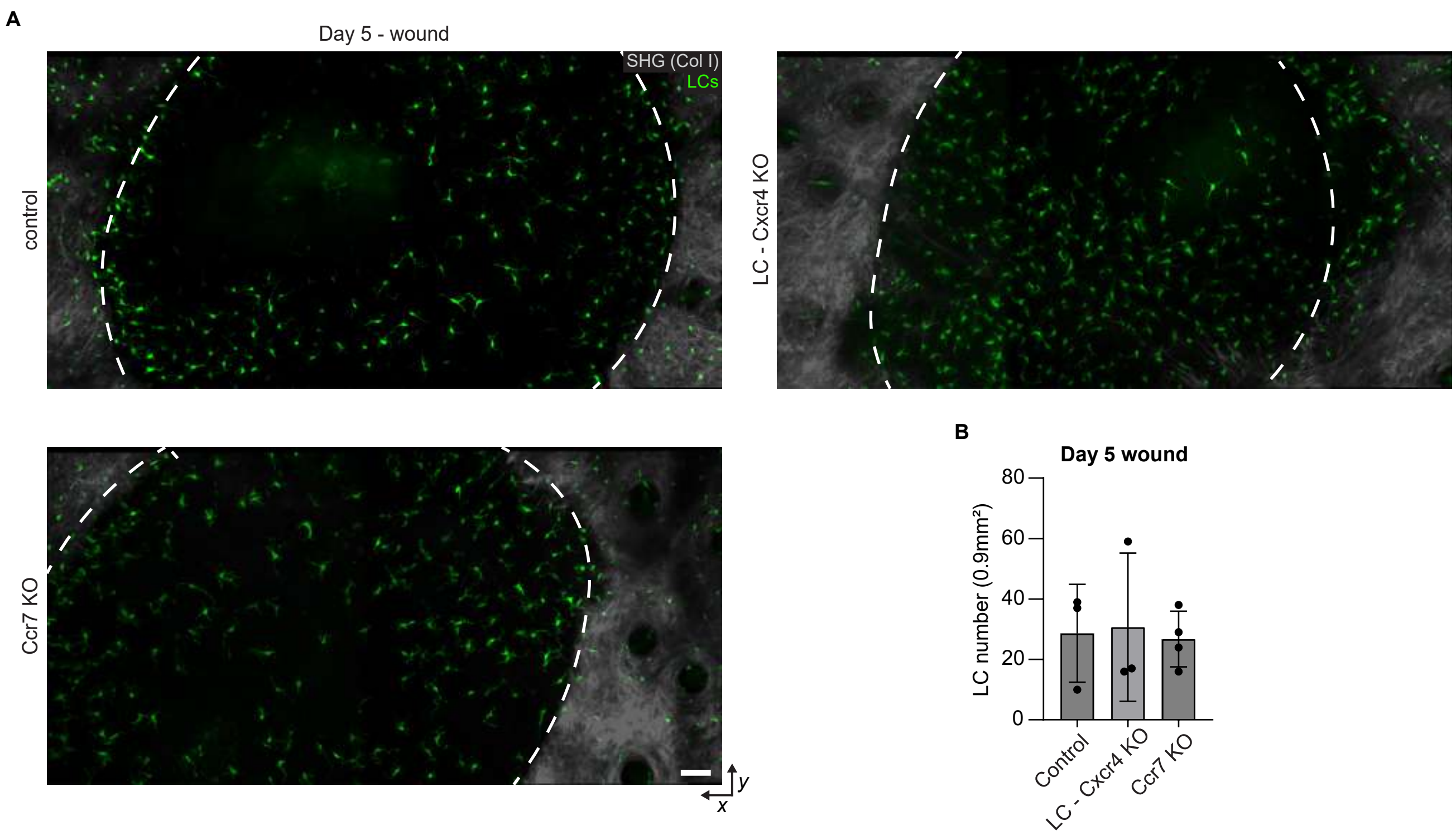

Supplementary Figure Legends

Figure S1. Epithelial cell migration on Day 2 post-injury. A, Experimental design for the longitudinal study of in-vivo wound healing. Revisit imaging (camera icon) was performed on Day 0 and 5. Timelapse imaging (video icon) was performed on Day 2. **B**, Schematic of wound healing multiphoton intravital microscopy. **C**, Imaris track analysis of epithelial cells (**Figure 1B**) 2 days after wound induction. Colors project time (blue, 0h; red, 6h). *Top*: *x-y* view. *Bottom*: *x-z* view. Representative image from 3 mice. Scale bars, 100 µm. **D**, Zoomed migration tracks from **C**. The green frame is from the wound leading-edge epithelial migration zone, and the teal frame is from the epithelial proliferation zone[3]. Representative image from 3 mice. Scale bars, 100 µm. **E**, Mean total displacement of individual epithelial cells tracks over 6h plotted as a function of distance from the wound. Dashed line, initial wound boundary. Data analyzed using paired two-way ANOVA; $n = 3$ mice; data are mean ± s.d. with each dot representing individual mice. *** $P < 0.001$, **** $P < 0.0001$.

Figure S2. Tamoxifen-induced CreER recombination in vivo. A, The schematic illustrates the experimental design for CreER-mediated recombination, including the timing of tamoxifen injection and subsequent in vivo imaging in mice, such as in Epi-*Rac1* (*K14-CreER;Rac1*$^{-/- \text{ or } +/-}$) and LC-*Rac1* (*huLangerin-CreER Rac1*$^{-/- \text{ or } +/+}$), dual-labeling mouse line (*huLangerin-CreER; Rosa-stop-tdTomato; Lang-EGFP; K14-H2B-mCherry*). Revisit imaging (camera icon) was performed on Day 0 and 5. Timelapse imaging (video icon) was performed on Day 2. **B**, *In-vivo* microscopy images show *x-y* view of epithelial cells (red nuclei) in the epidermis of control and Epi-*Rac1* KO mice at 0, 5 and 8 days after wound induction. Dashed line indicates initial wound boundary or scab. Representative image from 3 mice per group. Scale bars, 100 µm. **C**, Stereomicroscopy imaging of control and Epi-*Rac1* KO mice at Day 0 and 8 wound healing. Representative image from 3 mice per group. Scale bars, 100 µm.

Figure S3. The majority of MHC-II^{+} cells in the wound epidermis are Langerhans cells. A, Confocal immunofluorescent images of MHC-II+ Langerhans cells at the wound epidermis at days 7, 13 and week 3. *Left:* Images show *x-y* view of MHC-II+ cells (green), Langerhans cells (red, CD207), and cell nuclei (blue, DAPI). *Right*: green and red composite and zoomed view. Dashed line indicates initial wound boundary. Representative images from 3 mice. Scale bars, 100 µm. **B**, Percentage of Langerhans cells (CD207+) positive for MHC-II+ in the epidermis during homeostasis and days 7, 13, and week 3 of wound healing. Data analyzed using unpaired one-way ANOVA; $n = 3$ mice; data are mean ± s.d. with each dot representing individual mice.

Figure S4. IL-34 is increased on Day 7 of wound healing. A, ELISA assay of CSF1 present at the wound site on Day 7 of healing compared to homeostasis. **B**, ELISA assay of IL-34 present at the wound site on Day 7 of healing compared to homeostasis. **C**, ELISA assay of CSF1 present at Day 7 of wound healing in the epidermis compared and dermis. **D**, ELISA assay of IL-34 present at Day 7 of wound

healing in the epidermis compared and dermis. **A-D**, Data analyzed using unpaired two-tailed *t*-test; $n = 4$ mice; data are mean ± s.d. with each dot representing individual mice. * $P < 0.05$.

Figure S5. Langerhans cells quickly recover their normal distribution in the epidermal basal layer after wound induction. **A**, *In-vivo* microscopy images of epithelial cells (red nuclei) and LCs (green) in the epidermis 10 days after wound induction. The wound and far zones are as designated in (**Figure 3A**). The dashed line separates the basal and granular layers of the epidermis. The solid line separates the epidermis and dermis. *Left*: *xz* and *xy* views of the wound epidermis. *Right*: *xy* and *xz* views of the far epidermis. Representative images from 6 mice. Scale bars, 50 µm. **B-C**, Timeline of LC vertical distribution in the epidermis during wound healing at the wound (**B**) and far (**C**) zones. Data quantified as the minimal distance to the basement membrane (solid line). $n = 6$ mice. **D-E**, Timeline of LC horizontal distribution in the epidermis during wound healing at the wound (**D**) and far (**E**) zones. Data quantified as the average minimum distance to 3 closest LCs. $n = 6$ mice. **B-E,** Representative quantification of LC distribution. Data analyzed using paired one-way ANOVA; data are mean ± s.d. with data points representing individual cells. **** $P < 0.0001$.

Figure S6. scRNA-Seq heterogeneity in the epidermis on Day 11 after wound induction. **A**, UMAP of scRNA-Seq data enriched for LCs collected from the ear epidermis 11 days after wound induction. LC clusters were identified by high *Langerin* (*Cd207*) expression and were divided into activated (high Cd80), steady-state (low Cd80), cycling (Top2a), migrating (Ccr7), and apoptotic (Ddias) categories. Other clusters were identified according to their top DEGs. **B**, Violin plots of scRNA-Seq data after light quality control showing each cluster's total read counts, feature gene counts, and percentage of mitochondrial genes expressed. **C**, *Left*: Violin plots showing the expression of cluster-defining genes across each cluster. *Right*: Feature plots showing the expression of the gene mentioned in the same row. Mono, Monocytes. Act, activated. Ss, steady-state. Cyc, cycling. Mig, migrating. Apop, apoptotic. KC, keratinocytes (epithelial cells). Neut, neutrophils. T-mem, T memory cells.

Figure S7. eLC subclustering and gene expression of genes related to GO terms. **A**, UMAP of monocyte and Langerhans cells re-clustering, highlighting eLC subclusters. eLC were subclustered into steady-state (low *Cd80*, high *Cdh1*) and activated (high *Cd80*, low *Cdh1*). **B**, *Left*: violin plots showing *Cd80* and *Cdh1* expression in eLC subclusters. *Right*: Feature plots showing the expression of the gene mentioned in the same row. Data analyzed using an unpaired *t*-test. **** $P < 0.0001$. **C-F**, Dot plots showing the expression of genes in (Fig. 5g) GO terms according to clusters: Mono (**C**), pre-mLC (**D**), mLC (**E**), and eLC (**F**). Highlighted color shades match genes to their corresponding GO term.

Figure S8. Adoptive transfer of monocytes give rise to Langerhans cells during wound healing. **A**, The schematic shows the experimental design for adoptive transfer of monocytes to test their differentiation into Langerhans cells during wound healing. **B**, Flow cytometry gating strategy for the enrichment of bone marrow harvested monocytes. **C**, Mean percentage of monocytes harvested before and after MACS isolation. Data analyzed using unpaired two-tailed *t*-test; $n = 3$ mice; data are mean ± s.d. with each dot representing individual mice. **** $P < 0.0001$. **D**, Confocal immunofluorescent images of

donor monocytes-derived Langerhans cells at the wound epidermis 14 days after wound induction. *Left*: Images show *x-y* view of LCs (green, CD207), donor origin (red, CD45.2), and cell nuclei (blue, DAPI). *Right*: zoomed view in composite, green channel only, and red channel only. Dashed line indicates initial wound boundary. Representative images from 3 mice. Scale bars, 100 µm.

Figure S9. Characterization of epidermal CCR2-GFP cells at Day 13 of wound healing. A, Flow cytometry gating strategy for the characterization of CCR2-GFP myeloid cells in the epidermis at Day 11 of wound healing. **B**, Mean percentages of epidermal LyC6+, Ly6C+CD207+, and CD207+ cells in CCR2-GFP negative and positive myeloid cells at Day 11 of wound healing. Data analyzed using unpaired two-way ANOVA; $n = 4$ mice; data are mean ± s.d. with each dot representing individual mice. * $P < 0.05$.

Figure S10. CXCR4 and CCR7 are not required for Langerhans cell migration during re-epithelialization. A, *In-vivo* microscopy images show *x-y* view of dermis SHG collagen (gray) and LCs (green) in the epidermis 5 days after wound induction. Dashed line indicates initial wound boundary. *Top left*: control mouse. *Top right*: *LC-Cxcr4 KO* mouse. *Bottom*: *Ccr7 KO* mouse. Representative images are shown. n = 3 control, n = 3 LC-Cxcr4 KO, and n = 4 Ccr7 KO mice. Scale bar, 50 µm. **B**, Mean LC number inside the wound epidermis from control, LC-*Cxcr4 KO*, and *Ccr7 KO* mice. Imaging was performed 5 days after wound induction. Wound area quantified 0.09 mm^2 per mouse. Data analyzed using unpaired one-way ANOVA; n = 3 control, n = 3 LC-Cxcr4 KO, and n = 4 Ccr7 KO mice; data are mean ± s.d. with each dot representing individual mice.

Supplementary Video Legends

Video S1. Day 2 epithelial and LC migration. Time-lapse recording over 6h of epithelial cells (red nuclei) and LCs (green) 2 days post-wound induction (PWI). Dashed line, initial wound boundary. Solid line, basal membrane separating epidermis from dermis. *Top*: *x-y* view. *Bottom*: *x-z* view shows the epidermis (red) and dermis (collagen SHG, blue). Representative video from 3 mice.

Video S2. Epi-*Rac1* KO and control mice Day 2 epithelial and LC migration. Time-lapse recording over 6h in *x-y* view of epithelial cells (red nuclei) and LCs (green) 2 days post-wound induction (PWI). *Top*: control mouse. *Bottom*: Epi-*Rac1 KO* mouse. Dashed line, initial wound boundary. Representative video from 3 mice.

Video S3. In vivo LC proliferation within the wound epidermis at Day 7 post injury. Time-lapse recording over 5h in *x-y* view of proliferative LCs (green) at 7 days post-wound induction (PWI). Dermis SHG collagen is shown in gray. White arrows indicate diving LCs. Dashed line, initial wound boundary. Representative video from 3 mice. Scale bar, 50 µm.

Video S4. Zoom of LC proliferation on Day 7 post injury. Zoomed-in example of proliferative LCs (green) in **Video S3**. Representative video from 3 mice. Scale bar, 10 µm.

Video S5. Day 2 epithelial cell and LC migration in Cxcr2-inhibited and control mice. Time-lapse recording over 6h in *x-y* view of epithelial cells (red nuclei) and LCs (green) 2 days post-wound induction (PWI). *Top*: control mouse 1% DMSO. *Bottom*: drug-treated mouse CXCR2 (Danirixin). Dashed line, initial wound boundary. Representative video from 3 mice.
